# No accelerated progression of subclinical atherosclerosis with integrase strand transfer inhibitors compared to non-nucleoside reverse transcriptase inhibitors

**DOI:** 10.1093/jac/dkae383

**Published:** 2024-10-25

**Authors:** Javier García-Abellán, José A García, Sergio Padilla, Marta Fernández-González, Vanesa Agulló, Paula Mascarell, Ángela Botella, Félix Gutiérrez, Mar Masiá

**Affiliations:** Infectious Diseases Unit, Hospital General Universitario de Elche and Universidad Miguel Hernández de Elche, Alicante, Spain; CIBER de Enfermedades Infecciosas (CIBERINFEC), Instituto de Salud Carlos III, Madrid, Spain; Infectious Diseases Unit, Hospital General Universitario de Elche and Universidad Miguel Hernández de Elche, Alicante, Spain; CIBER de Enfermedades Infecciosas (CIBERINFEC), Instituto de Salud Carlos III, Madrid, Spain; Infectious Diseases Unit, Hospital General Universitario de Elche and Universidad Miguel Hernández de Elche, Alicante, Spain; CIBER de Enfermedades Infecciosas (CIBERINFEC), Instituto de Salud Carlos III, Madrid, Spain; CIBER de Enfermedades Infecciosas (CIBERINFEC), Instituto de Salud Carlos III, Madrid, Spain; Infectious Diseases Unit, Hospital General Universitario de Elche, Alicante, Spain; Infectious Diseases Unit, Hospital General Universitario de Elche, Alicante, Spain; Infectious Diseases Unit, Hospital General Universitario de Elche and Universidad Miguel Hernández de Elche, Alicante, Spain; Infectious Diseases Unit, Hospital General Universitario de Elche, Alicante, Spain; Infectious Diseases Unit, Hospital General Universitario de Elche and Universidad Miguel Hernández de Elche, Alicante, Spain; CIBER de Enfermedades Infecciosas (CIBERINFEC), Instituto de Salud Carlos III, Madrid, Spain; Infectious Diseases Unit, Hospital General Universitario de Elche and Universidad Miguel Hernández de Elche, Alicante, Spain; CIBER de Enfermedades Infecciosas (CIBERINFEC), Instituto de Salud Carlos III, Madrid, Spain

## Abstract

**Background:**

The role of integrase strand transfer inhibitors (INSTI) in the cardiovascular risk of people with HIV is controversial.

**Objectives:**

To assess the association of INSTI to subclinical atherosclerosis progression measured with the carotid intima-media thickness (cIMT).

**Methods:**

Prospective study in virologically suppressed people with HIV receiving INSTI- or NNRTI-based regimens. cIMT was measured at baseline, 48 and 96 weeks. cIMT progression was analysed both as a continuous and categorical variable, defined as cIMT increase ≥ 10% and/or new carotid plaque. Adjustments through Cox proportional hazard regression and linear mixed models, and propensity score matching were conducted.

**Results:**

190 participants were recruited and 173 completed the 96 week follow-up. 107 (56.3%) were receiving an INSTI-containing, 128 (67.4%) a NNRTI-containing and 45 (23.7%) a NNRTI plus an INSTI-containing regimen. The overall median (IQR) 2-year change of cIMT was 0.029 (−0.041 to 0.124) mm; 87 (45.8%) participants experienced a cIMT increase ≥ 10%, of whom 54 (28.4%) developed a new carotid plaque. Adjusted Cox regression showed no differences between INSTI and NNRTI groups in the categorical 2-year progression of cIMT, both including or excluding participants receiving INSTI + NNRTI. Similar results were observed for the continuous cIMT increase through adjusted linear mixed models. Propensity score matching showed no significant differences in the 2 year cIMT change between treatment groups [0.049 mm (−0.031–0.103) in the INSTI group versus 0.047 mm (−0.023–0.115) in the NNRTI group; *P* = 0.647]. cIMT progression was associated with traditional cardiovascular risk factors.

**Conclusions:**

INSTI-based regimens are not associated with increased progression of subclinical atherosclerosis when compared to NNRTI.

## Introduction

Cardiovascular disease has emerged as one of the primary causes of morbidity and mortality in people with HIV (PWH).^1^ The risk of myocardial infarction and cerebrovascular disease in PWH is 2-fold higher compared to the general population.^2,3^ Among the contributing pathogenic mechanisms specifically linked to HIV infection, certain antiretroviral drugs have been implicated, predominantly protease inhibitors and abacavir.^4,5^

Contemporary ART relies on integrase strand transfer inhibitors (INSTI). In initial studies comparing PI- and INSTI-based regimens, INSTI were shown to have a lesser impact on lipid and cardiovascular biomarker levels, both in ART-naive individuals and in switching studies.^6,7^ However, accumulating data suggest that initiating or switching to INSTI could be associated with a higher risk of unfavourable metabolic outcomes, such as weight gain, increases in blood pressure and an enhanced risk of hypertension or diabetes.^8–11^ Furthermore, recent evidence suggests a higher incidence of cardiovascular disease in PWH receiving INSTI-based regimens.^12^ Nevertheless, the metabolic effects and cardiovascular risks linked with INSTI have not shown consistency across studies, and additional research has yielded conflicting results.^13–15^ Assessing the influence of INSTI on the dynamics of atherosclerosis might contribute to expanding our understanding about the cardiovascular impact of this class of ART.

Carotid intima-media thickness (cIMT) assessed by B-mode ultrasonography is a non-invasive imaging modality that allows the detection of asymptomatic atherosclerotic vascular disease.^16^ The evaluation of cIMT has proven valuable in assessing the accelerated progression of subclinical atherosclerosis in PWH.^17^ Furthermore, cIMT is a predictor of future cardiovascular events,^18^ and of mortality within the HIV population.^19,20^ The accessibility, low toxicity and low cost, make cIMT measurement a suitable option for repeated measurements to assess atherosclerosis progression.

We conducted a prospective study in a contemporary cohort of virologically suppressed PWH and assessed the contribution of INSTI to subclinical atherosclerosis progression measured with the cIMT.

## Methods

### Study design, participants and study procedures

A prospective study was carried out at Hospital General Universitario de Elche, Spain. All participants were adults (> 18 years old) with HIV, enrolled between 26 October 2017 and 18 February 2019, and were followed-up until 12 January 2022. Participants included had been receiving ART with regimens based on NNRTI or INSTI, with undetectable viral load (HIV-1 RNA levels < 50 copies/mL) during at least 6 months before inclusion in the study. Participants with known cardiovascular disease, those on treatment with a PI-containing regimen, pregnant women or those becoming pregnant during follow-up, were excluded from the study.

Participants were followed-up for 2 years with face-to-face visits scheduled at baseline, 48 and 96 weeks. On each visit, physical examination and anthropometric measures including blood pressure, weight, height and BMI were collected, and blood samples were obtained for biochemical, metabolic and virological measurements. Blood samples were processed and plasma was obtained and cryopreserved at −80°C. Lymphocyte counts and their subsets were measured in fresh EDTA whole blood (AQUIOS CL Flow Cytometer, Beckman Coulter).

### Subclinical atherosclerosis measurement

Carotid artery intima-media thickness measurement was carried out at baseline, 48- and 96-week visits by a single trained ultrasonographer blinded to patients’ ART, following a standardized protocol, as previously described.^21,22^ B-mode ultrasound of carotids was performed using a Toshiba system (Toshiba Aplio 400), equipped with a 7–12 MHz linear array transducer. Ultrasonic scans were recorded from right and left common carotid, internal carotid artery and bifurcation. Measurements in millimetres from these six locations, using semiautomated detection software, were averaged and reported as a single cIMT (6-point cIMT).

We analysed the increase of cIMT both as a continuous variable and also as a categorical variable, defining cIMT progression as a 6-point cIMT increase ≥ 10% and/or detection of new carotid plaque (any measurement of cIMT > 1.5 mm).

### Inmuno-activation, inflammation and prothrombotic biomarkers

High-sensitivity C-reactive protein (hsCRP) was measured by immunoturbidimetric assay (CRP Gold Latex, DiAgam, Belgium) with an automated instrument (VITROS^®^ 5600 System, Ortho Clinical Diagnostics). D-dimer [Human D2D (D-Dimer) ELISA Kit], soluble ICAM-1 (sICAM-1) [Human ICAM-1/CD54 (intercellular adhesion molecule 1) ELISA Kit], soluble CD14 (sCD14) [Human sCD14 (Soluble Cluster of Differentiation 14) ELISA Kit] and soluble CD163 (sCD163) [Human sCD163 (Soluble Cluster of Differentiation 163) ELISA Kit] were measured by enzyme-linked immunosorbent assay (ELISA Kits, Elabscience Biotechnology Inc., USA) with an automated instrument (Dynex DS2^®^ ELISA system).

### Statistical analyses

We defined 2-year change in cIMT as the difference between baseline and 96-week measures. Mann–Whitney–Wilcoxon or Student’s *t*-tests were used for group comparison in continuous variables, according to the result of Shapiro Wilk’s contrast of normality. For categorical variables, comparisons were performed using the χ^2^ for variables with two or more categories, and Fisher’s exact tests for dichotomous variables. For multiple simultaneous comparisons, a Bonferroni adjustment was applied. We used multivariate adjusted Cox proportional hazard regression models to assess the association of cardiovascular risk factors, HIV-related factors, inflammatory biomarkers and ART with cIMT progression defined as a categorical variable incorporating covariates of interest. As a secondary exploratory analysis, linear mixed models with a random term for patient were used to examine associations with cIMT increase as a continuous variable.

To emulate some of the characteristics of a randomized study to estimate the effects of the INSTI-based regimen on subclinical atherosclerosis, treatment groups were balanced through propensity score matching with caliper 0.1. The covariates for propensity matching included durations of exposure to each ART regimen, age, sex, Framingham risk score, cIMT at baseline and baseline CD4 T cells.

For the calculation of the statistical power of our study, we analysed the difference in the 2 year cIMT increase between INSTI- and NNRTI-based groups. Our primary hypothesis was that cIMT progression over 96 weeks would be higher in participants with INSTI-based regimens compared with those on NNRTI-based regimens. With sample sizes of 62 participants on INSTI and 83 on NNRTI, our study provided 80% power using a Mann–Whitney *U* or Wilcoxon rank-sum test to detect a 2 year difference in cIMT change of at least 0.0192 mm.

Statistical significance was defined at a *P* value of < 0.05, and for Bonferroni adjustment at *P* value of < 0.0025 for continuous variables and *P* value of < 0.003 for discrete variables. Statistical analyses were performed with R version 4.0.3 software (R Foundation for Statistical Computing).

### Ethics

The study was approved by the Ethical Committee of Hospital General Universitario de Elche, and all participants signed an informed consent.

## Results

### Participants’ characteristics

Of 190 participants recruited at baseline, 190 and 173 completed the follow-up at 48- and 96-week visits. Reasons for dropout during follow-up are shown in Figure S1 (available as Supplementary data at *JAC* Online).

Baseline participants’ characteristics are detailed in Table 1. Median (IQR) age at enrolment was 48 (39–54) years, 154 (81.1%) were male, 111 (59%) had at least one traditional cardiovascular risk factor, 95 (50.5%) were smokers, and 22 (11.7%), 19 (10%) and 5 (2.6%) participants had dyslipaemia, hypertension or diabetes, respectively. The median (IQR) 10-year Framingham and atherosclerotic cardiovascular disease (ASCVD) risk scores were 6.3 (3.7–14.5) % and 2.6 (1.3–7.1) %, respectively, and the median (IQR) BMI was 24.7 (22.5–27.8) kg/m^2^.

**Table 1.** Characteristics of patients according to the antiretroviral regimen composition

Antiretroviral regimen						
	All patients	INSTI-containing regimen	NNRTI-containing regimen	Both INSTI and NNRTI-containing regimen	*P* value^∗^	*P* value^∗∗^
Patients, no.	190 (100)	62 (32.6)	83 (43.7)	45 (23.7)	—	—
Male sex	154 (81.1)	46 (74.2)	69 (83.1)	39 (86.7)	0.217	0.579
Age, years	48 (39–54)	49 (42–55)	49 (42–54)	39 (33–51)	0.900	0.164
Baseline CVD risk factors, no. (%)						
Any traditional CVD risk factor^a^	111 (59)	37 (60.7)	49 (60)	25 (55.6)	1.000	0.882
≥2 traditional CVD risk factors^a^	23 (12.2)	8 (13.1)	13 (16)	2 (4.4)	0.812	0.261
Current smoking	95 (50.5)	30 (49.2)	44 (53.7)	21 (46.7)	0.616	0.466
Hypertension	19 (10)	8 (13)	8 (9.6)	3 (6.7)	0.598	1.000
Dyslipidaemia	22 (11.7)	8 (13)	11 (13.4)	3 (6.7)	1.000	0.648
Diabetes	5 (2.6)	3 (4.8)	2 (2.4)	0 (0)	0.651	1.000
Framingham risk score, %	6.3 (3.7–14.5)	6.6 (3.5–17)	6.5 (4.7–14.6)	4.4 (2.7–9.4)	0.375	0.045
ASCVD risk score, %	2.6 (1.3–7.1)	2.4 (1–9.1)	4 (1.7–8.1)	1.8 (1–4.5)	0.281	0.030
Baseline cardio-metabolic parameters, no. (%)						
Blood pressure (BP), mm Hg						
Systolic	121 (111–134)	123 (111–131)	123 (111–136.5)	118 (111–129)	0.413	0.188
Diastolic	70 (64–77)	71 (66–77)	70 (63–79)	67 (63–76)	0.522	0.920
BMI, kg/m^2^	24.7 (22.5–27.8)	24.7 (22–27.8)	25.2 (22.6–27.7)	24.4 (23.1–27.5)	0.513	0.490
Weight, kg	73 (64–83)	71.5 (63–83)	73.5 (66–84)	75.5 (68–83.5)	0.351	0.741
Total cholesterol, mg/dL	171 (149–195)	167.5 (147–185)	180 (150–199)	170 (146–197)	0.057	0.067
LDL-cholesterol, mg/dL	102 (88–125)	97 (85–113)	110 (93–131)	99 (82–124)	0.004	0.006
HDL-cholesterol, mg/dL	46 (38–54)	45 (35–56)	44 (38–53)	48 (44–54)	0.905	0.222
Triglycerides, mg/dL	98 (71–137)	96 (74–153)	101 (73–135)	83 (61–117)	0.770	0.349
Baseline inflammatory biomarkers						
hsCRP, ng/mL	0.7 (0.3–2)	0.5 (0.3–1.9)	0.6 (0.3–1.4)	0.8 (0.3–2.3)	0.628	0.939
D-dimer, pg/mL	0.3 (0.2–0.5)	0.4 (0.3–0.7)	0.3 (0.2–0.5)	0.4 (0.2–0.5)	0.020	0.041
sCD14, pg/mL	5034 (4445–5670)	5033 (4346–5668)	5037 (4550–5738)	5056 (4382–5383)	0.665	0.474
sCD163, pg/mL	249 (182–328)	293 (175–396)	256 (196–326)	223 (171–286)	0.414	0.669
sICAM-1, pg/mL	82 (65–107)	89 (71–113)	76 (62–102)	802 (66–97)	0.076	0.144
Baseline immunovirological status and ART						
CD4 T cell count nadir, cells/mm^3^	250 (139–351)	212 (80–339)	250 (155–312)	300 (178–417)	0.383	0.768
CD4 T cell count, cells/mm^3^	687 (496–866)	690 (433–868)	688 (501–866)	665 (533–852)	0.647	0.693
CD4 T cell percentage, %	36 (30–42)	35.5 (28–41)	35.3 (30–41)	38 (32–42)	0.544	0.894
CD4/CD8 ratio	0.9 (0.7–1.3)	0.8 (0.6–1.3)	0.9 (0.7–1.3)	1 (0.8–1.3)	0.232	0.529
Months of exposure to last ART	33 (19–49)	27.1 (17–38)	49.9 (28–63)	21.4 (11–37)	0.001	0.001
Baseline cIMT, mm	0.808 (0.72–1.03)	0.818 (0.75–1.06)	0.848 (0.72–1.04)	0.773 (0.69–0.92)	0.987	0.333
Two year changes in body weight, BP and cIMT during the study						
Body weight increase, kg	1 (−1.5–4.5)	1 (−1.1–4.2)	1.4 (−1.8–4.6)	1.5 (−0.5–4.5)	0.866	0.853
Systolic BP increase, mmHg	0.1 (−12–10.5)	−3.5 (−14.8–9.8)	0.5 (−9–11)	1 (−10–9)	0.270	0.398
cIMT increase, mm^b^	0.029 (−0.041–0.124)	0.044 (−0.040–0.142)	0.021 (−0.048–0.115)	0.024 (0.027–0.085)	0.415	0.597
cIMT progression, %^c^	87 (45.8)	30 (48.8)	39 (47)	18 (40)	1.000	0.884

Categorical variables are expressed as number (percentage) and continuous variables as median (IQR).

INSTI, integrase strand transfer inhibitors; CVD, cardiovascular disease; ASCVD, atherosclerotic cardiovascular disease; BP, blood pressure; hsCRP, high-sensitivity C-reactive protein; sCD14, soluble CD14; sCD163, soluble CD163; sICAM-1, soluble intercellular adhesion molecule-1; cIMT, carotid intima-media thickness.

^a^Traditional CVD risk factors: current smoking, hypertension, dyslipidaemia and diabetes.

^b^cIMT increase represents the difference in mm between median cIMT at baseline and median cIMT at 96-week.

^c^cIMT progression was defined as a 6-point cIMT increase ≥ 10% and/or detection of new carotid plaques (any measurement of cIMT > 1.5 mm) during the 96-week follow-up.

^∗^ *P* value for the comparison between participants of the INSTI-containing regimen versus NNRTI-containing regimen

^∗∗^ *P* value for the comparison between participants with INSTI-containing regimen versus non-INSTI-containing regimen.

Among the study participants, 172 (90.5%) had maintained the same ART combination for at least 1 year, and 15 (7.9%) had switched to the current treatment regimen within the previous 6 months. The median (IQR) duration of the latter ART regimen at baseline was 33 (19–49) months. Regarding ART composition at baseline, 128 (67.4%) participants were receiving a NNRTI-containing regimen and 107 (56.3%), an INSTI-containing regimen, of whom 45 (23.7%) were receiving a combination of a NNRTI plus an INSTI. Detailed information about the specific INSTI and NNRTI used is summarized in Table S1. The median (IQR) duration of exposure to INSTI at baseline was 27.1 (17–38) months and to NNRTI 49.9 (28–63) months. During the 96-week follow-up, 10 participants (5.26%) switched to an INSTI-containing regimen, with a median duration of exposure since drug change of 18 (6–22) months. In 27.4% of participants receiving an INSTI, it was their first ART regimen.

Table 1 shows the baseline characteristics of participants according to ART group. Treatment group comparisons used an intention-to-treat approach. In the comparison of participants with INSTI- against participants with NNRTI-containing regimen, the INSTI group showed lower levels of LDL-cholesterol (*P* = 0.004), total cholesterol (*P* = 0.057), higher levels of D-dimer (*P* = 0.020) and fewer months of suppressed viral load at baseline (*P* = 0.002). However, the prevalence of traditional cardiovascular risk factors was not different among groups. There were no differences in the increase in blood pressure or body weight between treatment groups over the study period. Participants on INSTI-based regimens (including those on INSTI plus NNRTI) had lower Framingham and ASCVD risk scores than those treated with non-INSTI regimens, but no differences were observed in the scores when the group receiving INSTI plus NNRTI was excluded.

### Two-year progression in carotid artery intima-media thickness

The overall median (IQR) 2-year change of cIMT was 0.029 (−0.041 to 0.124) mm. A total of 87 (45.8%) participants experienced an increase in the 6-point cIMT ≥ 10%, with 54 (28.4%) of them developing a new carotid plaque. No significant differences in the 2 year change of cIMT were observed in the unadjusted analysis between patients receiving INSTI-containing regimens and those receiving NNRTI-containing regimens (Table 1). Similarly, no differences between ART groups were observed in the progression of cIMT defined as a categorical variable.

**Figure 1.**
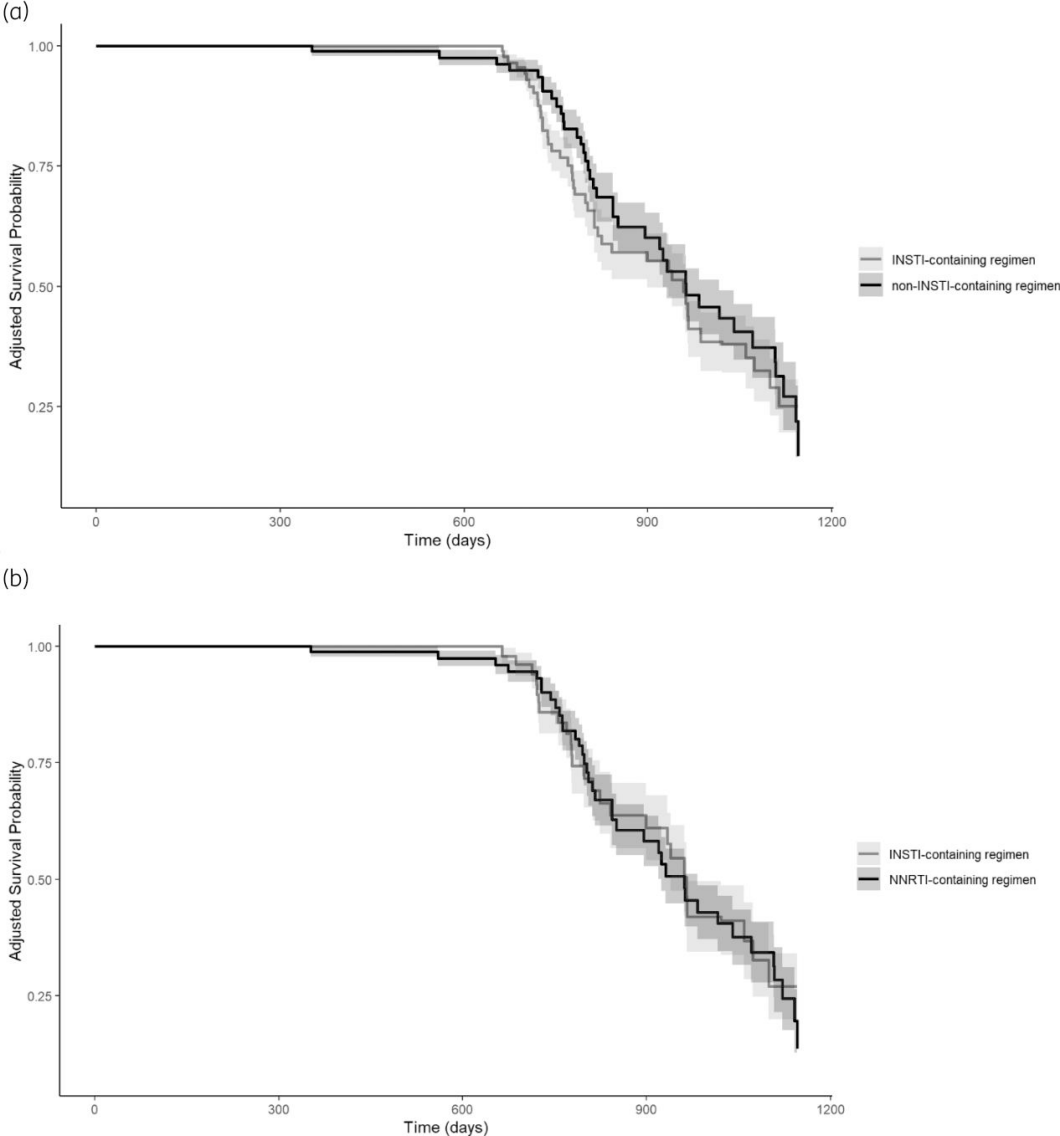
Kaplan–Meier curves to estimate the cumulative proportion of patients developing cIMT progression according to the antiretroviral regimen. (a) Participants with INSTI-containing versus non-INSTI-containing regimen (participants on INSTI plus NNRTI are included in the INSTI group). (b) Participants with INSTI-containing versus NNRTI-containing regimen (participants on INSTI plus NNRTI are excluded from the INSTI group).

Table 2 shows the baseline characteristics of the participants categorized by cIMT progression. In the univariate analysis, age (*P* < 0.001), systolic (*P* < 0.001) and diastolic blood pressure (*P* < 0.001), BMI (*P* = 0.044), cardiovascular risk indexes [Framingham score (*P* < 0.001) and ASCVD score (*P* < 0.001)], HDL-cholesterol (*P* = 0.013), LDL-cholesterol (*P* = 0.026), triglycerides (*P* = 0.005), D-dimer (*P* = 0.037) and sCD14 (*P* = 0.040) were associated with cIMT progression. Among HIV-related factors, participants experiencing cIMT progression showed a lower CD4 T cell percentage (*P* = 0.047). No significant differences between groups were observed in historical exposures to ABC (36.8% of participants experiencing cIMT progression versus 30.1%; *P* = 0.356, with a median duration of exposure of 2.7 versus 2.1 years; *P* = 0.357) or to protease inhibitors (39.1% of participants experiencing cIMT progression versus 29.1%; *P* = 0.167, with a median duration of exposure of 10.9 versus 10.5 years; *P* = 0.941). A multivariate Cox proportional hazards regression model was carried out adjusting for variables showing statistically significant difference between cIMT progression groups and covariates of interest, specifically age, sex, INSTI-based ART, cIMT at baseline, and the values at each visit of systolic blood pressure, BMI, LDL-cholesterol, HDL-cholesterol, CD4 T cell percentage, hsCRP, sCD14 and D-dimer. Results showed that age, systolic blood pressure, HDL-cholesterol, LDL-cholesterol, CD4 T cell percentage, D-dimer and sCD14 were independent factors associated with cIMT progression. INSTI-exposure showed a non-significant trend to significance (*P* = 0.094). When the participants receiving ART with an INSTI plus a NNRTI were excluded from the analysis, no relationship was observed between INSTI and cIMT progression (Table 2). Kaplan–Meier curves show the adjusted probability of cIMT progression during follow-up among participants with INSTI- versus non-INSTI-containing regimens (Figure 1a and b).

**Table 2.** Characteristics of patients according to the progression of cIMT during the 2 year follow-up

cIMT progression^b^							
	Yes		No		*P*-value	Adjusted HR^c^	Adjusted HR^d^
Patients, no.	87	(45.8)	103	(54.2)	—	—	—
Male sex	71	(81.6)	83	(80.6)	1.000	1.24(0.93–1.64)	1.19(0.86–1.65)
Age, years	53	(46–58)	44	(36–51)	0.001	1.03(1.02–1.04)	1.03(1.01–1.04)
Baseline CVD risk factors, no. (%)							
Any traditional CVD risk factor^a^	55	(63.2)	56	(55.4)	0.301		
≥2 traditional CVD risk factors^a^	15	(17.2)	8	(7.9)	0.073		
Current smoking	43	(49.4)	52	(51.5)	0.884		
Hypertension	13	(14.9)	6	(5.8)	0.051		
Dyslipidaemia	17	(19.5)	5	(5)	0.003		
Diabetes	3	(3.4)	2	(1.9)	0.662		
Framingham risk score, %	11	(5.5–20.3)	4.7	(3.1–8.1)	0.001		
ASCVD risk score, %	5.9	(2.4–11)	1.8	(1–3.7)	0.001		
Baseline cardio-metabolic parameters, no. (%)							
Blood pressure (BP), mm Hg							
Systolic	127	(114–138)	118	(110–126)	0.001	1.01(1.00–1.01)	1.01(1.01–1.02)
Diastolic	73	(66–80)	67	(62–74)	0.001		
BMI, kg/m^2^	25.6	(22.7–28.2)	24	(22–27.4)	0.044	0.99(0.96–1.02)	1.04(1.00–1.08)
Weight, kg	73	(66–84)	71	(63–81)	0.119		
Total cholesterol, mg/dL	177	(151–199.5)	167	(147–186)	0.115		
LDL-cholesterol, mg/dL	110	(90.5–132)	99	(87–116)	0.026	1.01(1.00–1.01)	1.00(1.00–1.01)
HDL-cholesterol, mg/dL	43	(36–51)	48	(40–57)	0.013	0.98(0.97–0.99)	0.99(0.98–1.00)
Triglycerides, mg/dL	107	(73–170.5)	89	(69.2–123)	0.005		
Baseline inflammatory biomarkers							
hsCRP, ng/mL	0.9	(0.3–2.5)	0.6	(0.2–1.4)	0.072	1.10(0.91–1.34)	1.17(0.93–1.45)
D-dimer, pg/mL	0.4	(0.2–0.7)	0.3	(0.2–0.4)	0.037	1.57(1.19–2.08)	1.12(0.80–1.57)
sCD14, pg/mL	5232	(4640–5870)	4887	(4341–5366)	0.040	1.02(1.01–1.03)	1.02(1.01–1.04)
sCD163, pg/mL	259.2	(192.9–332)	240.9	(169.4–310)	0.299		
sICAM-1, pg/mL	84	(68.4–110.3)	80.4	(63.3–96.3)	0.236		
Baseline immunovirological status and ART							
CD4 T cell count nadir, cells/mm^3^	246	(142–388)	253	(142–388)	0.388		
CD4 T cell count, cells/mm^3^	654	(450–876)	711	(544–866)	0.286		
CD4 T cell percentage, %	34	(29–41)	38	(32–42)	0.047	0.99(0.98–1.00)	0.99(0.98–1.00)
CD4/CD8 ratio	0.9	(0.7–1.3)	0.9	(0.7–1.3)	0.342		
Months of exposure to last ART	33	(19–52)	33	(18–44)	0.369		
NNRTI treatment at baseline, no (%)	55	(63.2)	69	(67)	0.761		
INSTI treatment at baseline, no (%)	48	(55.2)	59	(57)	0.884	1.20(0.97–1.50)	0.98(0.75–1.27)
Baseline cIMT, mm	0.895	(0.754–1.152)	0.763	(0.688–0.893)	0.001	1.02(0.75–1.39)	1.18(0.85–1.62)
Two-year changes in body weight, BP and cIMT during the study							
Body weight increase, kg	3	(1–5.2)	2	(0.5–4.8)	0.275		
Systolic BP increase, mmHg	14	(4–20)	8	(1.2–14)	0.018		

cIMT, carotid intima-media thickness; CVD, cardiovascular disease; ASCVD, atherosclerotic cardiovascular disease; BP, blood pressure; hsCRP, high-sensitivity C-reactive protein; sCD14, soluble CD14; sCD163, soluble CD163; sICAM-1, soluble intercellular adhesion molecule-1; cIMT, carotid intima-media thickness; INSTI, integrase strand transfer inhibitors.

^a^Traditional CVD risk factors: current smoking, hypertension, dyslipidaemia and diabetes.

^b^cIMT progression was defined as a 6-point cIMT increase ≥ 10% and/or detection of new carotid plaques (any measurement of cIMT > 1.5 mm).

^c^Multivariate Cox proportional hazards regression model of factors associated with progression of cIMT > 10% and/or detection of new carotid plaque, adjusted for variables showing statistically significant difference between cIMT progression groups and covariates of interest, specifically age, sex, INSTI-based ART, cIMT at baseline, and the values at each visit of systolic blood pressure, BMI, LDL-cholesterol, HDL-cholesterol, CD4 T cell percentage, hsCRP, sCD14 and D-dimer.

^d^Multivariate Cox proportional hazards regression model of factors associated with progression of cIMT > 10% and/or detection of new carotid plaque and excluding from the analysis participants receiving ART with an INSTI plus a NNRTI, adjusted for variables showing statistically significant difference between cIMT progression groups and covariates of interest, specifically age, sex, INSTI-based ART, cIMT at baseline, and the values at each visit of systolic blood pressure, BMI, LDL-cholesterol, HDL-cholesterol, CD4 T cell percentage, hsCRP, sCD14 and D-dimer.

Factors associated with cIMT increase analysed as a continuous variable are shown in Table 3. In the univariate analysis, cIMT increase was associated with age, presence of two or more traditional cardiovascular risk factors, dyslipidaemia, higher levels of total, HDL- and LDL-cholesterol, higher levels of D-dimer and higher cIMT at baseline. A multivariate analysis through linear mixed model, including the significant variables associated with cIMT increase and the covariates of interest (age, sex, INSTI-containing ART, cIMT at baseline, presence of two or more cardiovascular risk factors at baseline, and the values at each visit of systolic blood pressure, BMI, LDL-cholesterol, HDL-cholesterol, CD4 T cell percentage, hsCRP, sCD14 and D-dimer), showed that HDL-cholesterol, LDL-cholesterol and D-dimer were independent factors associated with cIMT increase, and sex and cIMT at baseline were close to significance. No association was found with INSTI exposure. The same results concerning the relationship of INSTI with cIMT increase were observed when patients receiving an INSTI plus a NNRTI were excluded from the analysis (Table 3).

**Table 3.** Univariate and multivariate analysis of factors associated with cIMT increase analysed as a continuous variable during the 2 year follow-up

cIMT increase as a continuous variable				
Univariate analysis				
Variable	Regression coefficient Ω, × 10^−3^ mm (95% CI)	*P* value	Adjusted coefficient^b^, × 10^−3^	Adjusted coefficient^c^, × 10^−3^
Age at baseline, years	3.6 (0.5–6.7)	0.023^∗^	0.3 (−0.5–1.4)	0.4 (−0.6–1.3)
Male sex	15.2 (−70.0–100.4)	0.726	17.5 (−4.8–37.8)	21.5 (−7–33.5)
≥ 2 traditional CVD risk factors^a^ at baseline	106.5 (6.6–206.3)	0.037^∗^	−8.4 (−28.9–14.6)	−10.3 (−36.7–15.1)
Hypertension at baseline, no. (%)	69.7 (−39.3–178.7)	0.209	—	—
Systolic blood pressure (all values), mm Hg	0.3 (−0.1–0.7)	0.126	0.1 (−0.5–0.5)	0.1 (−0.4–0.6)
Systolic blood pressure increase, mm Hg	0.5 (−0.5–1.4)	0.345	—	—
Weight (all values), kg	0.2 (−0.3–0.7)	0.511	—	—
Weight gain. kg	1.8 (−2–5.5)	0.356	—	—
BMI at baseline, kg/m^2^	−1.2 (−10.9–8.5)	0.805	—	—
BMI (all values), kg/m^2^	0.6 (−1.3–2.4)	0.536	−0.3 (−2.6–2)	−0.3 (−2.5–2.3)
INSTI-containing regimen (intention to treat)	−36.2 (−105.3–33)	0.303	−4.1 (−16.6–13.4)	−4.2 (−22.5–11.6)
Dyslipidaemia at baseline, no. (%)	155.2 (52.7–257.7)	0.003^∗∗^	—	—
Total cholesterol (all values), mg/dL	0.3 (0.1–0.4)	0.002^∗∗^	—	—
HDL-cholesterol (all values), mg/dL	0.3 (0.1–0.7)	0.049	0.4 (0.1–0.8)	0.4 (0.1–0.8)
LDL-cholesterol (all values), mg/dL	0.3 (0.1–0.5)	0.009	0.2 (0.1–0.5)	0.3 (0.1–0.5)
Diabetes at baseline, no. (%)	7.5 (−196.9–212)	0.942	—	—
CD4 T cell percentage (all values), %	−0.3 (−0.2–4.1)	0.347	−0.1 (−1.1–0.9)	−0.4 (−1–0.7)
CD4 T cell count (all values), cells/mm^3^	0.1 (−0.1–0.2)	0.253	—	—
CD4/CD8 ratio (all values)	−3.4 (−1.6–97.0)	0.628	—	—
hsCRP (log, all values), ng/mL	11.5 (−0.1–23.2)	0.071	0.6 (−11.4–16.1)	−3.0 (−13.5–19.1)
D-dimer (log, all values), pg/mL	34.2 (10.9–57.4)	0.001^∗∗^	29.1 (6.7–50.3)	38.2 (6.5–50)
sCD14 (log, all values), pg/mL	75.5 (−9.5–158.2)	0.061	26.6 (−66.4–116.2)	−17.2 (−68.2–103.4)
Framingham risk score (all values), %	0.6 (−0.1–1.2)	0.107	—	—
ASCVD risk score (all values), %	1.3 (−1.3–9.5)	0.186	—	—
cIMT at baseline, mm	174.4 (75.6–273.1)	0.001^∗∗^	24 (−14.3–55.3)	25.3 (−2.9–49.5)

Ω Regression coefficients represent a greater or lesser change in carotid artery intima-media thickness (in millimetre) over 2 years per difference in the corresponding parameter.

cIMT, carotid intima-media thickness; INSTI, integrase strand transfer inhibitors; CV, cardiovascular; ASCVD, atherosclerotic cardiovascular disease; hsCRP, high-sensitivity C-reactive protein; sCD14, soluble CD14.

^a^Traditional CVD risk factors: current smoking, hypertension, dyslipidaemia and diabetes.

^b^Multivariate analysis through linear mixed model, including the significant variables associated with cIMT increase and the covariates of interest (age, sex, INSTI-containing ART, cIMT at baseline, presence of two or more cardiovascular risk factors at baseline, and the values at each visit of systolic blood pressure, BMI, LDL-cholesterol, HDL-cholesterol, CD4 T cell percentage, hsCRP, sCD14 and D-dimer). The analysis includes all participants.

^c^Multivariate analysis through linear mixed model, including the significant variables associated with cIMT increase and the covariates of interest (age, sex, INSTI-containing ART, cIMT at baseline, presence of two or more cardiovascular risk factors at baseline, and the values at each visit of systolic blood pressure, BMI, LDL-cholesterol, HDL-cholesterol, CD4 T cell percentage, hsCRP, sCD14 and D-dimer). The analysis excludes participants receiving an INSTI plus a NNRTI.

^∗^Significance thresholds for main analyses were set at *P* < 0.05 for continuous and discrete variables.

^∗∗^Significance thresholds for Bonferroni adjustment were set at *P* < 0.0025 for continuous variables and *P* < 0.003 for discrete variables.

To mitigate selection bias in assessing the impact of INSTI-based versus NNRTI-based regimens on cIMT progression, we conducted an adjusted analysis through propensity score matching. This approach excluded the participants receiving both an INSTI and a NNRTI. Using a caliper width of 0.1, two matched groups of 35 patients were selected, with similar distributions of the matching covariates age, sex, Framingham risk score, baseline CD4 T cell percentage, duration of exposure to each ART regimen, and cIMT measure at baseline (Table 4). In this analysis, no significant differences in categorical cIMT progression were observed (48.6% in the INSTI group versus 42.3% in the NNRTI group, *P* = 0.810). Similarly, no significant differences were observed in the 2-year change in cIMT between treatment groups [0.049 mm (−0.031–0.103) in the INSTI group versus 0.047 mm (−0.023–0.115) in NNRTI group; *P* = 0.647].

**Table 4.** Patients’ characteristics according to the antiretroviral regimen composition after propensity score matching

	INSTI-containing regimen		NNRTI-containing regimen		*P*–value	SMD effect size
Patients, no.	35	(50)	35	(50)	—	—
Male sex	29	(82.9)	26	(74.3)	0.561	0.140
Age, years	47	(42–55)	47	(42–54)	0.911	−0.072
Baseline CVD risk factors, no. (%)						
Any traditional CVD risk factor^a^	22	(64.7)	20	(57.1)	0.624	0.208
≥2 traditional CVD risk factors^a^	6	(17.6)	7	(20)	1.000	0.013
Current smoking	20	(58.8)	18	(51.4)	0.631	0.201
Hypertension	4	(11.4)	4	(11.4)	1.000	0.001
Dyslipidaemia	5	(14.3)	5	(14.3)	1.000	0.095
Diabetes	2	(5.7)	2	(5.7)	1.000	0.001
Framingham risk score, %	9.2	(2.8–18.2)	5.7	(3.8–12.5)	0.972	−0.016
ASCVD risk score, %	3.5	(1.4–9.2)	2.7	(1.4–6.6)	0.989	−0.043
Baseline cardio-metabolic parameters, no. (%)						
Blood pressure (BP), mm Hg						
Systolic	121	(110–134)	126	(114–139)	0.182	−0.308
Diastolic	72	(67–78)	70	(61–78)	0.553	0.118
BMI, kg/m^2^	25	(21.9–27.7)	25.2	(22.9–27.7)	0.435	−0.317
Weight, kg	73.5	(63–82.5)	74	(67.2–81.2)	0.707	−0.231
Total cholesterol, mg/dL	168	(148–179)	166	(152–207)	0.131	−0.385
LDL-cholesterol, mg/dL	97	(88–115)	110	(92–131)	0.077	−0.447
HDL-cholesterol, mg/dL	43	(37–52)	44	(37–55)	0.883	0.026
Triglycerides, mg/dL	92	(71–156)	98	(71–124)	0.911	0.200
Baseline inflammatory biomarkers						
hsCRP, ng/mL	0.3	(0.2–0.6)	0.6	(0.3–1.1)	0.015	0.047
D-dimer, pg/mL	0.3	(0.2–0.4)	0.4	(0.2–0.6)	0.658	0.174
sCD14, pg/mL	4896	(4103–5658)	5076	(4460–5789)	0.219	−0.357
sCD163, pg/mL	288	(176–335)	260	(196–316)	0.690	0.280
sICAM-1, pg/mL	84.1	(64–99)	75.2	(61–96)	0.384	0.273
Baseline immunovirological status and ART						
CD4 T cell count nadir, cells/mm^3^	220	(94–339)	252	(211–333)	0.369	0.086
CD4 T cell count, cells/mm^3^	732	(481–867)	600	(448–866)	0.577	0.250
CD4 T cell percentage, %	37.1	(31–42)	34	(32–41)	0.557	0.042
CD4/CD8 ratio	0.9	(0.7–1.3)	0.9	(0.7–1.2)	0.963	0.069
Months of exposure to last ART	32.8	(23.6–39.2)	39.9	(26.8–53.8)	0.102	−0.152
Baseline cIMT, mm	0.763	(0.726–0.978)	0.803	(0.713–0.940)	0.995	−0.126
Two year changes in body weight, BP and cIMT during the study						
Body weight increase, kg	1.5	(−0.1–4.5)	1.6	(−2.5–4.9)	0.441	0.100
Systolic BP increase, mmHg	−1	(−14–10.2)	2	(−8.5–18.5)	0.425	0.156
cIMT increase, mm^b^	0.049	(−0.031–0.103)	0.047	(−0.023–0.115)	0.647	−0.114
cIMT progression, %^c^	17	(48.6)	15	(42.3)	0.810	0.056

The covariates for propensity matching include age, sex, Framingham risk score, baseline CD4 T cell percentage, duration of exposure to each ART regimen, and cIMT at baseline. For this analysis, participants receiving both an INSTI and a NNRTI were excluded.

INSTI, integrase strand transfer inhibitors; SMD, standardized mean difference; CVD, cardiovascular disease; ASCVD, atherosclerotic cardiovascular disease; BP, blood pressure; hsCRP, high-sensitivity C-reactive protein; sCD14, soluble CD14; sCD163, soluble CD163; sICAM-1, soluble intercellular adhesion molecule-1; cIMT, carotid intima-media thickness.

^a^Traditional CVD risk factors: current smoking, hypertension, dyslipidaemia and diabetes

^b^cIMT increase represents the difference in mm between median cIMT at baseline and median cIMT at 96-week.

^c^cIMT progression was defined as a 6-point cIMT increase ≥ 10% and/or detection of new carotid plaques (any measurement of cIMT > 1.5 mm) during the 96-week follow-up.

## Discussion

In this contemporary cohort of virologically suppressed PWH on ART, exposure to INSTI-based regimens was not associated with increased progression of subclinical atherosclerosis when compared to NNRTI. We carried out multiple analyses and thorough adjustments for potential confounding factors, including propensity score matching to ensure balance between treatment groups, and results consistently revealed no significant relationship between INSTI use and enhanced cIMT progression. Our findings align with those reported in analyses conducted through target trial emulation in both the HIV-CAUSAL and the Antiretroviral Therapy Cohort Collaborations^14^ and the Swiss cohort,^13^ which did not confirm the increased risk of cardiovascular event development with INSTI-based regimens observed in the respond collaboration.^12^ As expected, our data support the pivotal role of traditional cardiovascular risk factors in the progression of atherosclerosis in PWH.

The role of INSTI in the cardiovascular risk of PWH is currently a topic of scientific interest and controversy, arising from emerging concerns regarding the cardio-metabolic complications associated with this class of ART. In this study, we assessed the effect of INSTI compared to NNRTI on the progression of atherosclerosis. NNRTI represent the second most common anchor drug class in contemporary ART regimens.^23,24^ This family of antiretroviral drugs has historically been associated with a relatively ‘benign’ impact on the cardiovascular system, which is attributed to its favourable metabolic profile, particularly exhibited by NVP and, to a lesser extent, RPV.^25–27^ Available evidence suggests that exposure to NNRTI, either as a class or through specific agents like NVP and EFV, is not associated with an increased risk of myocardial infarction.^28–30^ Similarly, exposure to NNRTI was associated in a prospective study with lower progression of cIMT compared to PI.^31^ Our results suggest no differences in subclinical atherosclerosis progression between INSTI and NNRTI drug families.

To assess the progression of subclinical atherosclerosis, our analysis incorporated the development of a new carotid plaque. Carotid plaque is a strong predictor of cardiovascular disease occurrence demonstrated in both the general and the HIV population, where it has been shown to improve the cardiovascular risk stratification.^32–36^ Accordingly, carotid plaque assessment using ultrasonography has been endorsed as a risk modifier/enhancer to reclassify cardiovascular risk in the guidelines on cardiovascular disease prevention in the HIV and the general population.^37,38^ Existing data about the relationship between regimens containing INSTI and subclinical atherosclerosis progression are currently scarce. In a randomized clinical trial including ART-naive participants, Stein *et al*. compared the cIMT progression in those initiating ART based on RAL, ATV/RTV or DRV/RTV.^39^ They did not find differences in cIMT change between antiretroviral regimens, with the exception of a lower cIMT progression at the carotid bifurcation in the ATV/RTV group. An observational prospective study analysed the cIMT increase over a 2-year period in 102 treatment-naive participants starting ART with TAF/FTC in combination with DTG, RAL or EVG.^40^ No control group with a different antiretroviral class was included. To our knowledge, no studies had compared the progression of subclinical atherosclerosis between INSTI- and NNRTI-based antiretroviral regimens.

Weight gain associated with INSTI represents one of the contributing factors through which this antiretroviral class may potentially increase the risk of cardiovascular disease. Several studies have documented increased weight gain associated with INSTI-based regimens compared to NNRTI-based regimens. This phenomenon has been observed in both ART-naive individuals^41–46^ and in those switching ART, primarily from regimens based on EFV.^47–50^ Follow-up has usually lasted 12–24 months.^42,48–50^ Noteworthy, the increase in weight with INSTI was not observed with treatment durations exceeding 2 years in the REPRIEVE trial.^51^ Consistent with the findings from the REPRIEVE, we observed no significant differences in weight changes between the INSTI- and NNRTI-based antiretroviral groups in our cohort, where the median duration of the antiretroviral regimens at baseline was over 2 years. Additionally, we did not observe a relationship between changes in weight and the progression of subclinical atherosclerosis. While excess weight is associated with greater risk for cardiovascular morbidity and mortality,^52^ and a higher BMI has been associated with increased cIMT in PWH in two cross-sectional studies in South Africa,^53,54^ some studies have neither observed an association between BMI or weight changes and cardiovascular events in the HIV population.^55,56^

During the 2-year follow-up period, a high proportion of participants in our cohort showed an increase in the cIMT, and a significant percentage developed new carotid plaques, a frequency similar to that described in other cohorts of PWH.^57,58^ As expected, the traditional cardiovascular risk factors emerged as the predominant factors associated with cIMT progression. Participants experiencing subclinical atherosclerosis progression showed a distinct lipid profile, characterized by lower levels of baseline HDL-cholesterol and higher levels of LDL-cholesterol during follow-up. The group receiving NNRTI-based regimens showed higher levels of LDL-cholesterol, which was included among adjusting variables. Our analysis revealed a significant association of hypertension and blood pressure increases with progression of atherosclerosis. Remarkably, we did not observe significant differences between treatment groups in the prevalence of hypertension or blood pressure changes. The existing evidence about the relationship between INSTI and blood pressure is limited and divergent. While several studies have reported a higher incidence of hypertension or blood pressure increases associated with INSTI- compared to NNRTI- and/or PI-containing regimens, both in ART-naive^8,11^ and in pre-treated PWH switching to another regimen,^9,10^ other studies have not observed such an association.^15,59^

In addition to traditional cardiovascular risk factors, chronic inflammation plays a critical role in the genesis and progression of atherosclerosis.^60^ PWH exhibit an enhanced pro-inflammatory state characterized by higher levels of several inflammation and coagulation markers, such as hsCRP and D-dimer, which strongly predict cardiovascular disease event development within this population.^61,62^ We observed a significant association of hsCRP and D-dimer levels with subclinical atherosclerosis progression in our cohort. However, we did not find differences in the levels of the biomarkers between participants receiving INSTI- and NNRTI-based regimens. Switch to INSTI from PI, NNRTI or enfuvirtide has been associated with a reduction in the levels of biomarkers of inflammation, insulin resistance and hypercoagulability.^63–66^ Likewise, treatment initiation with EVG/CBT versus EFV was associated with greater declines in hsCRP, sCD14 and Lp-PLA2.^67^ Of note, previous studies comparing biomarker changes with NNRTI versus INSTI primarily included EFV, whereas the predominant NNRTI administered in our cohort was RPV. EFV is associated with a more pro-atherogenic profile compared to RPV, primarily due to the lipid changes it induces.^68^ As far as we know, there is a lack of head-to-head studies directly comparing biomarker changes between RPV and INSTI.

The limitations of the study are primarily associated with the non-randomized assignment of the ART regimen. The sample size also represents a limitation, despite the study being powered to detect relatively small differences in cIMT progression between groups. Consequently, if there were an increased risk of subclinical atherosclerosis progression associated with INSTI, it is likely that the effect size would be small. Some of the participants receiving INSTI were concurrently taking NNRTI, which could have influenced the impact of INSTI on cIMT progression. However, the analyses including and excluding the subgroup of participants with an INSTI plus a NNRTI showed similar results. A proportion of participants in the cohort had been exposed to other antiretroviral families prior to inclusion in the study that might have also had an effect on cIMT. Strengths include the prospective study design, with longitudinal analysis of the cIMT progression during a 2-year follow-up, and the carotid measurement conducted by a single sonographer, who was blinded to the ART composition. Participants were pre-treated with antiretrovirals, thus enabling the analysis of the impact of each antiretroviral class on a stable condition. We used stringent criteria to define cIMT progression, aimed at identifying individuals with a higher likelihood of experiencing future cardiovascular events. This variable showed a strong association with traditional cardiovascular risk factors, which reinforces the reliability and consistency of the definition utilized. Finally, we conducted multiple analyses with adjustment for several potential confounders and found consistent results.

In conclusion, among PWH on stable ART, no evidence of accelerated progression of cIMT was observed with INSTI-based compared to NNRTI-based regimens. Instead, the traditional cardiovascular risk factors continue to play a central role in atherogenesis in PWH. Continued surveillance and investigation will contribute to fully understanding the cardiovascular impact of INSTI.

## Supplementary Material

dkae383_Supplementary_Data

## Supplementary data

Figure S1 and Table S1 are available as Supplementary data at *JAC* Online.

## References

[1] Weber MSR, Duran Ramirez JJ, Hentzien M et al Time trends in causes of death in people with HIV: insights from the Swiss HIV cohort study. Clin Infect Dis 2024; 79: 177–88. 10.1093/cid/ciae014 38214897 PMC11259222

[2] Shah ASV, Stelzle D, Lee KK et al Global burden of atherosclerotic cardiovascular disease in people living with HIV: systematic review and meta-analysis. Circulation 2018; 138: 1100–12. 10.1161/CIRCULATIONAHA.117.033369 29967196 PMC6221183

[3] Masiá M, Padilla S, García JA et al Evolving understanding of cardiovascular, cerebrovascular and peripheral arterial disease in people living with HIV and role of novel biomarkers. A study of the Spanish CoRIS cohort, 2004–2015. PLoS One 2019; 14: e0215507. 10.1371/journal.pone.0215507 31026289 PMC6485642

[4] Ryom L, Lundgren JD, El-Sadr W et al Cardiovascular disease and use of contemporary protease inhibitors: the D:A:D international prospective multicohort study. Lancet HIV 2018; 5: e291–300. 10.1016/S2352-3018(18)30043-2 29731407

[5] Jaschinski N, Greenberg L, Neesgaard B et al Recent abacavir use and incident cardiovascular disease in contemporary-treated people with HIV. AIDS 2023; 37: 467–75. 10.1097/QAD.0000000000003373 36001525

[6] Maggi P, Di Biagio A, Rusconi S et al Cardiovascular risk and dyslipidemia among persons living with HIV: a review. BMC Infect Dis 2017; 17: 551. 10.1186/s12879-017-2626-z 28793863 PMC5550957

[7] Clotet B, Feinberg J, van Lunzen J et al Once-daily dolutegravir versus darunavir plus ritonavir in antiretroviral-naive adults with HIV-1 infection (FLAMINGO): 48 week results from the randomised open-label phase 3b study. Lancet 2014; 383: 2222–31. 10.1016/S0140-6736(14)60084-2 24698485

[8] Galdamez R, García JA, Fernández M et al Short-term increase in risk of overweight and concomitant systolic blood pressure elevation in treatment-naïve persons starting INSTI-based antiretroviral therapy. Open Forum Infect Dis 2019; 6: ofz491. 10.1093/ofid/ofz491 32128334 PMC7047949

[9] Brennan AT, Nattey C, Kileel EM et al Change in body weight and risk of hypertension after switching from efavirenz to dolutegravir in adults living with HIV: evidence from routine care in Johannesburg, South Africa. EClinicalMedicine 2023; 57:101836. 10.1016/j.eclinm.2023.101836. 36816348 PMC9932660

[10] Summers NA, Lahiri CD, Angert CD et al Metabolic changes associated with the use of integrase strand transfer inhibitors among virally controlled women. J Acquir Immune Defic Syndr 2020; 85: 355–62. 10.1097/QAI.0000000000002447 33060420 PMC7577246

[11] Byonanebye DM, Polizzotto MN, Neesgaard B et al Incidence of hypertension in people with HIV who are treated with integrase inhibitors versus other antiretroviral regimens in the RESPOND cohort consortium. HIV Med 2022; 23: 895–910. 10.1111/hiv.13273 35233903 PMC9545382

[12] Neesgaard B, Greenberg L, Miró JM et al Associations between integrase strand-transfer inhibitors and cardiovascular disease in people living with HIV: a multicentre prospective study from the RESPOND cohort consortium. Lancet HIV 2022; 9: e474–85. 10.1016/S2352-3018(22)00094-7 35688166

[13] Surial B, Chammartin F, Damas J et al Impact of integrase inhibitors on cardiovascular disease events in people with human immunodeficiency virus starting antiretroviral therapy. Clin Infect Dis 2023; 77: 729–37. 10.1093/cid/ciad286 37157869 PMC10495132

[14] Rein SM, Lodi S, Logan RW et al Integrase strand-transfer inhibitor use and cardiovascular events in adults with HIV: an emulation of target trials in the HIV-CAUSAL collaboration and the antiretroviral therapy cohort collaboration. Lancet HIV 2023; 10: e723–32. 10.1016/S2352-3018(23)00233-3 37923486 PMC10695103

[15] Sempere A, Assoumou L, González-Cordón A et al Incidence of hypertension and blood pressure changes in persons with human immunodeficiency virus at high risk for cardiovascular disease switching from boosted protease inhibitors to dolutegravir: a post-hoc analysis of the 96-week randomised NEAT-022 trial. Clin Infect Dis 2023; 77: 991–1009. 10.1093/cid/ciad297. 37207617

[16] Iwakiri T, Yano Y, Sato Y et al Usefulness of carotid intima-media thickness measurement as an indicator of generalized atherosclerosis: findings from autopsy analysis. Atherosclerosis 2012; 225: 359–62. 10.1016/j.atherosclerosis.2012.10.033 23092826

[17] Hanna DB, Post WS, Deal JA et al HIV infection is associated with progression of subclinical carotid atherosclerosis. Clin Infect Dis 2015; 61: 640–50. 10.1093/cid/civ325 25904369 PMC4607734

[18] Lorenz MW, Markus HS, Bots ML et al Prediction of clinical cardiovascular events with carotid intima-media thickness: a systematic review and meta-analysis. Circulation 2007; 115: 459–67. 10.1161/CIRCULATIONAHA.106.628875 17242284

[19] Hanna DB, Moon JY, Haberlen SA et al Carotid artery atherosclerosis is associated with mortality in HIV-positive women and men. AIDS 2018; 32: 2393–403. 10.1097/QAD.0000000000001972 30102657 PMC6170701

[20] Mangili A, Polak JF, Quach LA et al Markers of atherosclerosis and inflammation and mortality in patients with HIV infection. Atherosclerosis 2011; 214: 468–73. 10.1016/j.atherosclerosis.2010.11.013 21130995 PMC3034311

[21] Touboul PJ, Hennerici MG, Meairs S et al Mannheim carotid intima-media thickness and plaque consensus (2004-2006-2011). an update on behalf of the advisory board of the 3rd, 4th and 5th watching the risk symposia, at the 13th, 15th and 20th European Stroke Conferences, Mannheim, Germany, 2004, Brussels, Belgium, 2006, and Hamburg, Germany, 2011. Cerebrovasc Dis 2012; 34: 290–96. 10.1159/000343145. 23128470 PMC3760791

[22] Lidón F, Padilla S, García JA et al Contribution of human herpesvirus 8 and herpes simplex type 2 to progression of carotid intima-media thickness in people living with HIV. Open Forum Infect Dis 2019; 6: ofz041. 10.1093/ofid/ofz041 30815506 PMC6386804

[23] Ruiz-Algueró M, Hernando V, Riero M et al Temporal trends and geographic variability in the prescription of antiretroviral treatments in people living with HIV in Spain, 2004–2020. J Clin Med 2022; 11: 1896. 10.3390/jcm11071896 35407504 PMC8999235

[24] Eaton EF, Tamhane A, Davy-Mendez T et al Trends in antiretroviral therapy prescription, durability and modification: new drugs, more changes, but less failure. AIDS 2018; 32: 347–55. 10.1097/QAD.0000000000001708 29194118 PMC6604808

[25] Vos AG, Venter WDF. Cardiovascular toxicity of contemporary antiretroviral therapy. Curr Opin HIV AIDS 2021; 16: 286–91. 10.1097/COH.0000000000000702 34545036

[26] Maggi P, Bellacosa C, Carito V et al Cardiovascular risk factors in patients on long-term treatment with nevirapine- or efavirenz-based regimens. J Antimicrob Chemother 2011; 66: 896–900. 10.1093/jac/dkq507 21393134

[27] Fisac C, Virgili N, Ferrer E et al A comparison of the effects of nevirapine and nelfinavir on metabolism and body habitus in antiretroviral-naive human immunodeficiency virus-infected patients: a randomized controlled study. J Clin Endocrinol Metab 2003; 88: 5186–92. 10.1210/jc.2002-021830 14602748

[28] Eyawo O, Brockman G, Goldsmith CH et al Risk of myocardial infarction among people living with HIV: an updated systematic review and meta-analysis. BMJ Open 2019; 9: e025874. 10.1136/bmjopen-2018-025874 PMC6773316 31551371

[29] Silverberg MJ, Leyden WA, Xu L et al Immunodeficiency and risk of myocardial infarction among HIV-positive individuals with access to care. J Acquir Immune Defic Syndr 2014; 65: 160–66. 10.1097/QAI.0000000000000009 24442222

[30] Lang S, Mary-Krause M, Cotte L et al Impact of individual antiretroviral drugs on the risk of myocardial infarction in human immunodeficiency virus-infected patients: a case-control study nested within the French hospital database on HIV ANRS cohort CO4. Arch Intern Med 2010; 170: 1228–38. 10.1001/archinternmed.2010.197 20660842

[31] Baker JV, Henry WK, Patel P et al Progression of carotid intima-media thickness in a contemporary human immunodeficiency virus cohort. Clin Infect Dis 2011; 53: 826–35. 10.1093/cid/cir497 21860012 PMC3174096

[32] Ihle-Hansen H, Vigen T, Berge T et al Carotid plaque score for stroke and cardiovascular risk prediction in a middle-aged cohort from the general population. J Am Heart Assoc 2023; 12: e030739. 10.1161/JAHA.123.030739 37609981 PMC10547315

[33] Wyman RA, Mays ME, McBride PE et al Ultrasound-detected carotid plaque as a predictor of cardiovascular events. Vasc Med 2006; 11: 123–30. 10.1191/1358863x06vm666ra 16886843

[34] Zanni MV, Abbara S, Lo J et al Increased coronary atherosclerotic plaque vulnerability by coronary computed tomography angiography in HIV-infected men. AIDS 2013; 27: 1263–72. 10.1097/QAD.0b013e32835eca9b 23324657 PMC3740057

[35] Janjua SA, Staziaki PV, Szilveszter B et al Presence, characteristics, and prognostic associations of carotid plaque among people living with HIV. Circ Cardiovasc Imaging 2017; 10: e005777. 10.1161/CIRCIMAGING.116.005777 29021257 PMC5679423

[36] Nambi V, Chambless L, Folsom AR et al Carotid intima-media thickness and presence or absence of plaque improves prediction of coronary heart disease risk: the ARIC (atherosclerosis risk in communities) study. J Am Coll Cardiol 2010; 55: 1600–7. 10.1016/j.jacc.2009.11.075 20378078 PMC2862308

[37] Visseren FLJ, Mach F, Smulders YM et al 2021 ESC Guidelines on cardiovascular disease prevention in clinical practice. Eur Heart J 2021; 42: 3227–337. 10.1093/eurheartj/ehab484 34458905

[38] Feinstein MJ, Hsue PY, Benjamin LA et al Characteristics, prevention, and management of cardiovascular disease in people living with HIV: a scientific statement from the American Heart Association. Circulation 2019; 140: e98–124. 10.1161/CIR.0000000000000695 31154814 PMC7993364

[39] Stein JH, Ribaudo HJ, Hodis HN et al A prospective, randomized clinical trial of antiretroviral therapies on carotid wall thickness. AIDS 2015; 29: 1775–83. 10.1097/QAD.0000000000000762 26372383 PMC4571277

[40] Calza L, Borderi M, Colangeli V et al No progression of subclinical atherosclerosis in HIV-infected patients starting an initial regimen including tenofovir alafenamide/emtricitabine plus raltegravir, dolutegravir or elvitegravir/cobicistat during a two-year follow-up. Infect Dis (Lond) 2020; 52: 249–56. 10.1080/23744235.2019.1707279 31876437

[41] Kanters S, Renaud F, Rangaraj A et al Evidence synthesis evaluating body weight gain among people treating HIV with antiretroviral therapy—a systematic literature review and network meta-analysis. EClinicalMedicine 2022; 48: 101412. 10.1016/j.eclinm.2022.101412 35706487 PMC9112095

[42] Sax PE, Erlandson KM, Lake JE et al Weight gain following initiation of antiretroviral therapy: risk factors in randomized comparative clinical trials. Clin Infect Dis 2020; 71: 1379–89. 10.1093/cid/ciz999 31606734 PMC7486849

[43] Venter WDF, Moorhouse M, Sokhela S et al Dolutegravir plus two different prodrugs of tenofovir to treat HIV. N Engl J Med 2019; 381: 803–15. 10.1056/NEJMoa1902824 31339677

[44] Bourgi K, Rebeiro PF, Turner M et al Greater weight gain in treatment-naive persons starting dolutegravir-based antiretroviral therapy. Clin Infect Dis 2020; 70: 1267–74. 10.1093/cid/ciz407 31100116 PMC8205610

[45] Bakal DR, Coelho LE, Luz PM et al Obesity following ART initiation is common and influenced by both traditional and HIV-/ART-specific risk factors. J Antimicrob Chemother 2018; 73: 2177–85. 10.1093/jac/dky145 29722811 PMC6054231

[46] Bourgi K, Jenkins CA, Rebeiro PF et al Weight gain among treatment-naïve persons with HIV starting integrase inhibitors compared to non-nucleoside reverse transcriptase inhibitors or protease inhibitors in a large observational cohort in the United States and Canada. J Int AIDS Soc 2020; 23: e25484. 10.1002/jia2.25484 32294337 PMC7159248

[47] Norwood J, Turner M, Bofill C et al Brief report: weight gain in persons with HIV switched from efavirenz-based to integrase strand transfer inhibitor-based regimens. J Acquir Immune Defic Syndr 2017; 76: 527–31. 10.1097/QAI.0000000000001525 28825943 PMC5680113

[48] Lake JE, Wu K, Bares SH et al Risk factors for weight gain following switch to integrase inhibitor-based antiretroviral therapy. Clin Infect Dis 2020; 71: e471–7. 10.1093/cid/ciaa177 32099991 PMC7713693

[49] Tse J, Prajapati G, Zhao X et al Weight gain following switch to integrase inhibitors from non-nucleoside reverse transcriptase or protease inhibitors in people living with HIV in the United States: analyses of electronic medical records and prescription claims. Curr Med Res Opin 2023; 39: 1237–46. 10.1080/03007995.2023.2239661 37480288

[50] Kerchberger AM, Sheth AN, Angert CD et al Weight gain associated with integrase stand transfer inhibitor use in women. Clin Infect Dis 2020; 71: 593–600. 10.1093/cid/ciz853 31504324 PMC7384314

[51] Kileel EM, Malvestutto CD, Lo J et al Changes in body mass index with longer-term integrase inhibitor use: a longitudinal analysis of data from the randomized trial to prevent vascular events in human immunodeficiency virus (REPRIEVE). Clin Infect Dis 2023; 76: 2010–13. 10.1093/cid/ciad107 36825498 PMC10474926

[52] Lopez-Jimenez F, Almahmeed W, Bays H et al Obesity and cardiovascular disease: mechanistic insights and management strategies. Eur J Prev Cardiol 2022; 29: 2218–37. 10.1093/eurjpc/zwac187 36007112

[53] Schoffelen AF, de Groot E, Tempelman HA et al Carotid intima media thickness in mainly female HIV-infected subjects in rural South Africa: association with cardiovascular but not HIV-related factors. Clin Infect Dis 2015; 61: 1606–14. 10.1093/cid/civ586 26215596

[54] Roozen GVT, Vos AG, Tempelman HA et al Cardiovascular disease risk and its determinants in people living with HIV across different settings in South Africa. HIV Med 2020; 21: 386–96. 10.1111/hiv.12831 31852030 PMC7318654

[55] Bares SH, Wu X, Tassiopoulos K et al Weight gain after antiretroviral therapy initiation and subsequent risk of metabolic and cardiovascular disease. Clin Infect Dis 2024; 78: 395–401. 10.1093/cid/ciad545 37698083 PMC10874261

[56] Pelchen-Matthews A, Ryom L, Borges ÁH et al Aging and the evolution of comorbidities among HIV-positive individuals in a European cohort. AIDS 2018; 32: 2405–16. 10.1097/QAD.0000000000001967 30134296

[57] Hsue PY, Scherzer R, Hunt PW et al Carotid intima-media thickness progression in HIV-infected adults occurs preferentially at the carotid bifurcation and is predicted by inflammation. J Am Heart Assoc 2012; 1: jah3-e000422. 10.1161/JAHA.111.000422 PMC3487373 23130122

[58] McLaughlin MM, Ma Y, Scherzer R et al Association of viral persistence and atherosclerosis in adults with treated HIV infection. JAMA Netw Open 2020; 3: e2018099. 10.1001/jamanetworkopen.2020.18099 33119103 PMC7596582

[59] Hatleberg CI, Ryom L, d'Arminio Monforte A et al Association between exposure to antiretroviral drugs and the incidence of hypertension in HIV-positive persons: the data collection on adverse events of anti-HIV drugs (D:A:D) study. HIV Med 2018; 19: 605–18. 10.1111/hiv.12639 30019813 PMC6169998

[60] Zanni MV, Grinspoon SK. HIV-specific immune dysregulation and atherosclerosis. Curr HIV/AIDS Rep 2012; 9: 200–5. 10.1007/s11904-012-0123-y 22638983

[61] Nordell AD, McKenna M, Borges ÁH et al Severity of cardiovascular disease outcomes among patients with HIV is related to markers of inflammation and coagulation. J Am Heart Assoc 2014; 3: e000844. 10.1161/JAHA.114.000844 24870935 PMC4309077

[62] Hsue PY, Tawakol A. Inflammation and fibrosis in HIV: getting to the heart of the matter. Circ Cardiovasc Imaging 2016; 9: e004427. 10.1161/CIRCIMAGING.116.004427 26951604 PMC5761657

[63] Martínez E, D'Albuquerque PM, Llibre JM et al Changes in cardiovascular biomarkers in HIV-infected patients switching from ritonavir-boosted protease inhibitors to raltegravir. AIDS 2012; 26: 2315–26. 10.1097/QAD.0b013e328359f29c 23018438

[64] González-Cordón A, Assoumou L, Moyle G et al Switching from boosted PIs to dolutegravir decreases soluble CD14 and adiponectin in high cardiovascular risk people living with HIV. J Antimicrob Chemother 2021; 76: 2380–93. 10.1093/jac/dkab158 34120186

[65] Silva EF, Charreau I, Gourmel B et al Decreases in inflammatory and coagulation biomarkers levels in HIV-infected patients switching from enfuvirtide to raltegravir: ANRS 138 substudy. J Infect Dis 2013; 208: 892–97. 10.1093/infdis/jit280 23801606

[66] Lake JE, McComsey GA, Hulgan T et al Switch to raltegravir decreases soluble CD14 in virologically suppressed overweight women: the women, integrase and fat accumulation trial. HIV Med 2014; 15: 431–41. 10.1111/hiv.12128 24506429 PMC4107004

[67] Hileman CO, Kinley B, Scharen-Guivel V et al Differential reduction in monocyte activation and vascular inflammation with integrase inhibitor-based initial antiretroviral therapy among HIV-infected individuals. J Infect Dis 2015; 212: 345–54. 10.1093/infdis/jiv004 25583168 PMC4539910

[68] Tebas P, Sension M, Arribas J et al Lipid levels and changes in body fat distribution in treatment-naive, HIV-1-infected adults treated with rilpivirine or efavirenz for 96 weeks in the ECHO and THRIVE trials. Clin Infect Dis 2014; 59: 425–34. 10.1093/cid/ciu234 24729492

