# Supplementary material for: No accelerated progression of subclinical atherosclerosis with integrase strand transfer inhibitors compared to non-nucleoside reverse transcriptase inhibitors
Source: J Antimicrob Chemother. 2024 Oct 25;80(1):126–37. doi: 10.1093/jac/dkae383 (PMC11695909; doi:10.1093/jac/dkae383)
Supplement: dkae383_Supplementary_Data [file dkae383_supplementary_data.docx]

**Figure S1. Flow chart of persons with HIV participating in the study.**


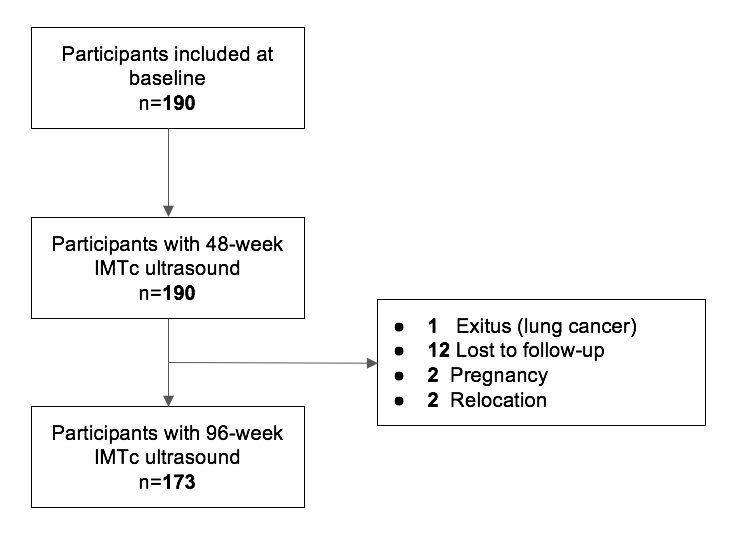


cIMT, carotid intima-media thickness

**Table S1. Antiretroviral agents included in the non-nucleoside reverse transcriptase inhibitors and integrase strand transfer inhibitors families during the two-year follow-up.**

| **Family of ART** | **Baseline** | **48-week visit** | **96-week visit** |
| --- | --- | --- | --- |
| **NNRTI, no.**  Doravirine  Efavirenz  Etravirine  Nevirapine  Rilpivirine  Rilpivirine long-acting | 1  14  1  6  84  22 | 1  9  0  6  78  28 | 2  6  0  5  72  24 |
| **INSTI, no.**  Bictegravir  Cabotegravir  Cabotegravir long-acting  Dolutegravir  Elvitegravir  Raltegravir | 0  4  22  67  5  9 | 1  1  28  68  6  6 | 1  4  24  68  6  5 |

INSTI, integrase strand transfer inhibitors.
